# A case of panspinal epidural abscess that presented with meningeal irritation

**DOI:** 10.1002/ams2.294

**Published:** 2017-07-06

**Authors:** Naoki Okada, Takashi Nishiyama, Maki Kurihara, Yusuke Nishimura, Yoshiro Nishimura, Yukihiro Ando, Saori Otsubo, Katsumi Yamada, Yuji Maeda

**Affiliations:** ^1^ Department of Disaster and Emergency Medicine Kobe University Graduate School of Medicine Kobe City Hyogo Japan

**Keywords:** bacterial meningitis, epidural abscess, magnetic resonance imaging, meningitis, psoas abscess

## Abstract

**Case:**

In rare cases, spinal epidural abscess involves the entire spine and can lead to neurological deficits and sepsis if treatment is delayed or suboptimal. A 65‐year‐old man was admitted with a diagnosis of bacterial meningitis. After admission, magnetic resonance imaging showed a spinal epidural abscess from the cervical to lumbar spine. Blood culture revealed *Staphylococcus aureus*. The patient was initially treated medically because he had no neurological deficits. Repeat blood culture remained positive and abscesses were found in the mediastinum and bilateral psoas muscles.

**Outcome:**

Surgery was carried out and the patient's postoperative course was satisfactory.

**Conclusion:**

Spinal epidural abscess can extensively affect the spine and may present with the symptoms of bacterial meningitis. It is essential to examine the entire spine and paraspinal regions and to treat early in cases of spinal epidural abscess.

## Introduction

Spinal epidural abscess (SEA) is a rare condition, but can lead to neurological deficits and sepsis if treatment is delayed or suboptimal.^1^ Spinal epidural abscess generally extends along several vertebrae, but in rare cases can involve the entire spine, resulting in so‐called panspinal or holospinal epidural abscess.^2^ We report a case of panspinal epidural abscess that presented with meningeal irritation.

## Case

**Figure 1:**
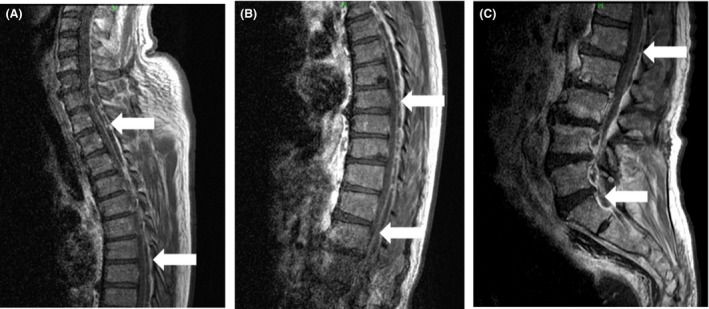
Magnetic resonance imaging with contrast on post‐admission day 4 in a 65‐year‐old male patient admitted with meningeal irritation. A cervical spinal epidural abscess dorsal to the spinal cord extends from C6 (A) (arrows) to the thoracic spine (B) (arrows). The abscess continues to the lumbar spine. Pyogenic spondylitis can be seen from L1 to S1 (C) (arrows).

**Figure 2:**
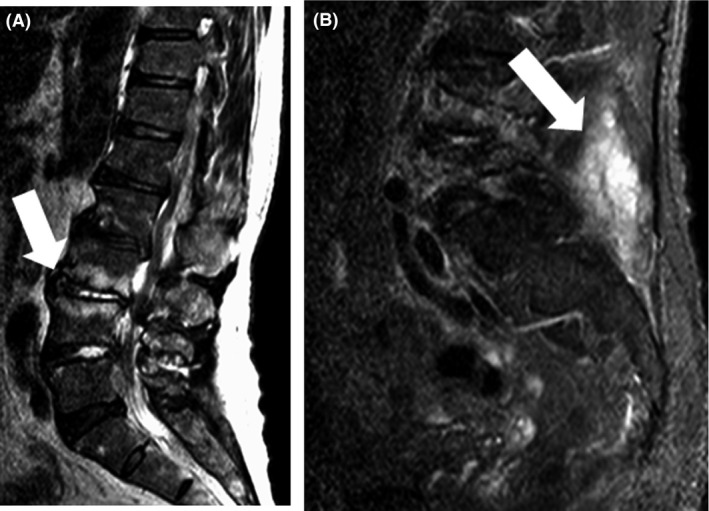
Magnetic resonance imaging on post‐admission day 4 in a 65‐year‐old man admitted with meningeal irritation. A, Pyogenic spondylitis is seen from L1 to S1 (arrows). B, Inflammation in connective tissue is seen in the dorsal lumbar region (arrows).

**Figure 3:**
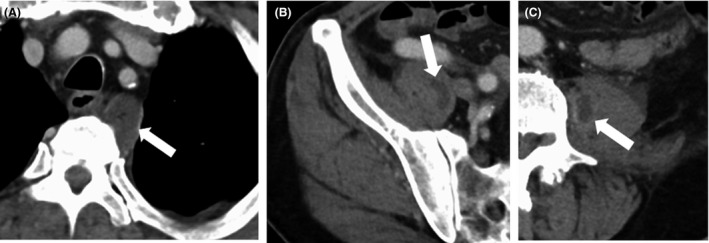
Computed tomography with contrast on post‐admission day 6 in a 65‐year‐old man admitted with meningeal irritation. Abscesses are seen in the mediastinum (A) (arrows) and bilateral psoas muscles (B) (arrows).

A 65‐year‐old man was brought to hospital by ambulance and admitted to our emergency department with fever and brief impairment of consciousness. This condition followed 4 days of general fatigue and appetite loss. The patient had a history of lumbar canal stenosis and had undergone sacral epidural block 2 weeks previously. He had no other predisposing factors for infection, such as diabetes, alcoholism, or i.v. drug use. On arrival, the patient's vital signs were as follows: Glasgow Coma Scale, E4V5M6; blood pressure, 134/80 mmHg; pulse rate, 136 b.p.m.; body temperature, 38.6°C; respiration rate, 25 breaths/min; and oxygen saturation, 100% in room air. On physical examination, the patient presented with neck stiffness. A knock pain was positive in the right lumbar region. He showed no radiculopathy or neurological deficits. Laboratory testing revealed a white blood cell count of 8500/μL and C‐reactive protein level of 24 mg/dL. Cranial computed tomography was unremarkable. Cerebrospinal fluid (CSF) was sampled at the L2–3 level to avoid the region of lumbar canal stenosis and epidural block. Blood culture was also carried out. The CSF was xanthochromic with elevated protein (948 mg/dL), polymorphonuclear pleocytosis (282/μL), and low glucose (72 mg/dL; plasma glucose, 176 mg/dL). We diagnosed bacterial meningitis on the basis of fever, meningeal irritation, and polycytosis in the CSF. We administered meropenem and vancomycin. Blood culture revealed that the pathogen was methicillin‐sensitive *Staphylococcus aureus*. We changed antibiotics to ceftriaxone on day 2. Blood culture on day 4 remained positive. Spinal epidural abscess or pyogenic spondylitis was suspected because of the patient's recent sacral epidural block. Magnetic resonance imaging showed a dorsal epidural abscess extending from C6 to S1 (Fig. 1). Spondylitis was also present from L1 to S1 (Fig. 2A). Inflammation was observed in the connective tissue in the dorsal lumbar region (Fig. 2B). We informed the patient that the sacral epidural block was the likely cause of SEA. Initially, we planned conservative therapy because the patient had no neurological deficits. However, fever had been present for 7 days, the white blood cell count had increased, and blood culture remained positive on days 5 and 7. Furthermore, computed tomography with contrast confirmed abscesses in the mediastinum (Fig. 3A) and bilateral psoas muscles on day 6 (Fig. 3B, C). Medical management was thus considered to be ineffective. Therefore, on day 8, the patient underwent epidural drainage by laminectomy at T2–T3, T10–T11, and L4–5, followed by mediastinal abscess drainage with video‐assisted thoracoscopy. The drainage was purulent. The postoperative diagnosis was panspinal epidural abscess, pyogenic spondylitis at L4–5, and mediastinal abscess. The patient's postoperative course was satisfactory. The patient received i.v. antibiotics for 8 weeks and was transferred to another hospital on day 61.

## Discussion

The findings of this case have two important clinical implications. First, SEA can affect the entire spine and may be accompanied by paraspinal abscesses. Spinal epidural abscess has an incidence of 1 per 10,000 hospital admissions; panspinal epidural abscess is rare.^2^, ^3^ There are only 15 previous reports of panspinal epidural abscess.^3^ In our patient, SEA affected C6 to S1; pyogenic spondylitis was also present from L1 to S1. These findings indicated panspinal epidural abscess. The epidural space can be infected by hematogenous spread (50%), direct extension from adjacent infection (33%), and spinal procedures (15%).^4^ The risk factors of SEA are diabetes, alcoholism, HIV, spinal abnormality or intervention, and potential local or systemic source of infection, such as epidural analgesia.^2^ Panspinal epidural abscess has been associated with diabetes, intra‐abdominal complications from Crohn's disease, immunosuppression from chemotherapy, psoas abscess, and neonatal sacral teratoma.^5^ Panspinal epidural abscess affects patients of all ages and should be suspected in high‐risk patients with back pain, fever, and neurological deficits.^2^ *Staphylococcus aureus* is the causative agent in two‐thirds of cases.^2^ The duration of antibiotic therapy is generally 6–8 weeks because vertebral osteomyelitis is present in most patients with SEA, whereas 4 weeks of antibiotics is generally recommended for bacterial meningitis.^2^, ^6^ A previous study reported that 12 of 15 patients with panspinal epidural abscess underwent surgery.^5^ Two patients who underwent surgery had rapid neurological decline and one died.^5^ The most important predictor of neurological outcome is preoperative neurological status.^2^, ^4^, ^7^ Early surgical intervention before deterioration of neurological function is needed to improve patient outcomes.^4^, ^5^, ^6^, ^7^ As for the operative indications for SEA, another study reported that diabetes, leukocytosis greater than 12,500/μL, positive blood cultures, and C‐reactive protein greater than 11.5 mg/dL are risk factors for failure of medical therapy. The presence of two risk factors elevates the failure rate to 40.2%; three or more risk factors elevates it to 76.9%.^8^ Our patient had three risk factors. He had no neurological deficits, but fever and positive blood culture persisted and paraspinal abscesses in the mediastinum and psoas muscles were found. Because of the risk of neurological deterioration and sepsis, we pursued surgical intervention. For patients with panspinal epidural abscess, undertaking decompressive laminectomy along the entire spine is impractical. Therefore, less extensive surgery, such as limited or skip laminectomy, is considered.^2^

The second clinical implication of this study is that SEA may present with meningeal irritation. The classical clinical triad of SEA comprises back pain, fever, and neurological deficits. However, this triad is present in only a minority of patients with SEA.^9^ Approximately 50% of patients with SEA are initially misdiagnosed.^1^ High protein levels and pleocytosis are observed in 75% of patients with SEA, and CSF culture results are positive in <25% of patients whose CSF is microbiologically assessed.^10^ Infection that originates in the spinal epidural space can extend locally or through the bloodstream to other sites.^2^ Abscesses in the mediastinum and bilateral psoas muscles were confirmed in our case. Epidural infection such as SEA and parameningial inflammation must be excluded in cases with positive CSF findings.

## Conclusion

Spinal epidural abscess can extensively affect the spine in severe cases and can present with symptoms of bacterial meningitis. Examining the entire spine and paraspinal regions and early treatment before deterioration of neurologic function are essential in cases of panspinal epidural abscess.

## Disclosure

Informed Consent: All informed consent was obtained from the subject and guardians.

Conflict of Interest: None declared.
